# Ryanodine-receptor-driven intracellular calcium dynamics underlying spatial association of synaptic plasticity

**DOI:** 10.1007/s10827-015-0579-z

**Published:** 2015-10-24

**Authors:** Daiki Futagi, Katsunori Kitano

**Affiliations:** Graduate School of Information Science and Engineering, Ritsumeikan University, 1-1-1 Nojihigashi, Kusatsu, Shiga 525-8577 Japan; Department of Human and Computer Intelligence, Ritsumeikan University, 1-1-1 Nojihigashi, Kusatsu, Shiga 525-8577 Japan

**Keywords:** Calcium signaling, Intracellular, Ryanodine receptor, Simulation

## Abstract

Synaptic modifications induced at one synapse are accompanied by hetero-synaptic changes at neighboring sites. In addition, it is suggested that the mechanism of spatial association of synaptic plasticity is based on intracellular calcium signaling that is mainly regulated by two types of receptors of endoplasmic reticulum calcium store: the ryanodine receptor (RyR) and the inositol triphosphate receptor (IP_3_R). However, it is not clear how these types of receptors regulate intracellular calcium flux and contribute to the outcome of calcium-dependent synaptic change. To understand the relation between the synaptic association and store-regulated calcium dynamics, we focused on the function of RyR calcium regulation and simulated its behavior by using a computational neuron model. As a result, we observed that RyR-regulated calcium release depended on spike timings of pre- and postsynaptic neurons. From the induction site of calcium release, the chain activation of RyRs occurred, and spike-like calcium increase propagated along the dendrite. For calcium signaling, the propagated calcium increase did not tend to attenuate; these characteristics came from an all-or-none behavior of RyR-sensitive calcium store. Considering the role of calcium dependent synaptic plasticity, the results suggest that RyR-regulated calcium propagation induces a similar change at the synapses. However, according to the dependence of RyR calcium regulation on the model parameters, whether the chain activation of RyRs occurred, sensitively depended on spatial expression of RyR and nominal fluctuation of calcium flux. Therefore, calcium regulation of RyR helps initiate rather than relay calcium propagation.

## Introduction

Synaptic modification is essential for developing and maintaining functional neural circuits and depends on neuronal activity (Bliss and Collingridge 1993; Katz and Shatz 1996; Martin et al. 2000). Results of physiological studies focusing on the dependence of synaptic plasticity on temporal activity of neurons have demonstrated that synaptic efficacy can either be long-term potentiated (LTP) or depressed (LTD) depending on firing rate (Bliss and Lomo 1973; Sjöström et al. 2001) and relative spike timing (STDP) (Markram et al. 1997; Bi and Poo 1998) of pre- and postsynaptic neurons. In addition to the temporal aspect of synaptic plasticity, some physiological studies showed that synaptic modification induced at one synapse is often accompanied by collective synaptic changes at neighboring sites in a postsynaptic dendrite (Bi 2002). The spatial synaptic plasticity association has also been observed in a global area: for example, CA1 and CA3 of the hippocampus (Lynch et al. 1977; Bradler and Barrionuevo 1989; Nishiyama et al. 2000), cortex (Hirsch et al. 1992), and amygdala (Royer and Paré 2003). This evidence implies that individual synapses are not independent, and the outcome of synaptic modification is partly determined by the association between synapses. The spatial aspect of synaptic plasticity provides the possibility to construct and stabilize selectivity of synaptic input that functionally activates neuronal circuits. According to a recent study, stimulus selectivity evolves by clustering and segregating synapses on dendrites by synchronized synaptic input (Takahashi et al. 2012). Therefore, revealing a mechanism of synaptic plasticity association will contribute to understanding the link between spatiotemporal input patterns, synaptic modulation, and formation of a functional circuit.

Although exact spatial synaptic association has not been clarified, it is assumed to be attributed to intracellular calcium signaling. Within a cell, many enzymes and proteins are affected by calcium ions, which are involved in multiple molecular processes that are relevant to modulation of cell function (Berridge et al. 2000). Over the years, molecular studies have shown that increasing postsynaptic calcium is required for the induction of bidirectional synaptic change. Increase in postsynaptic calcium concentration generally triggers two types of molecular signaling processes for the induction of synaptic change: one process mediates LTP by the action of protein kinases and the other process mediates LTD by the action of phosphatases (Colbran and Brown 2004; Munton et al. 2004). Based on experimental knowledge, it has been hypothesized that the outcome of synaptic change is mainly determined by the magnitude of postsynaptic calcium concentration (Johnston et al. 2003; Graupner and Brunel 2012; Hulme et al. 2012). The pathways that increase postsynaptic calcium concentration in cytoplasm are generally categorized into two types: one is calcium influx through NMDA receptors (NMDARs) and voltage-dependent calcium channels (VDCCs) in the membrane and the other is calcium release from intracellular calcium stores, such as the endoplasmic reticulum (ER). Regarding the former pathway, it has been suggested that calcium influx via NMDAR and VDCC is necessary for the induction of synaptic plasticity (Nevian and Sakmann 2006). Concerning the latter pathway, the calcium release from the ER is mediated by calcium-dependent receptors. Over the years, it has been shown that the calcium release is relevant to the induction of bidirectional homo-synaptic change (Reyes and Stanton 1996; Raymond and Redman 2002). In addition, recent physiological studies implicate that calcium store regulates wave-like intercellular calcium propagation (Watanabe et al. 2006) and the outcome of collective synaptic changes at neighboring sites (Nishiyama et al. 2000). Combined with the experimental suggestions that the outcome of synaptic plasticity depends on postsynaptic calcium concentration, it is hypothesized that calcium propagation regulated by the ER plays an important role in the mechanism of hetero-synaptic association.

The stored calcium ions in the ER are mainly released via two types of receptors: ryanodine receptor (RyR) and inositol 1,4,5-trisphosphate receptor (IP_3_R). Binding with calcium ions activates both types of receptors and induces calcium release; increased cytoplasm calcium concentration induces further calcium release from store. In addition, the magnitude of calcium release via IP_3_R depends on cytoplasmic IP_3_ concentrations (Bezprozvanny et al. 1991). On the other hand, RyR does not depend on IP_3_ and RyR-regulated calcium release can be all or none (Usachev and Thayer 1997). The combination of these receptors with their different characteristics likely regulates intracellular calcium signaling (Rose and Konnerth 2001). However, the role of these receptors in calcium regulation and the link between ER-calcium regulation and the outcome of collective synaptic change is not clear. In particular, compared with IP_3_R, the role of RyR calcium regulation is not well known. Some studies suggest that RyR is not necessary for the triggering and calcium propagation along the dendrite (Nakamura et al. 1999; Watanabe et al. 2006). In contrast, the results of some physiological studies suggest that RyR is expressed in the dendritic shaft (Seymour-Laurent and Barish 1995; Khodakhah and Armstrong 1997; Hertle and Yeckel 2007) and regulates calcium flux in postsynaptic dendrites (Futatsugi et al. 1999; Fujii et al. 2000). Thus, the specific role of RyR remains to be elucidated. Therefore, in this study, we focused on RyR and assessed the behavior of RyR-mediated spatiotemporal calcium flux. For the observation, we simulated intracellular calcium dynamics at a cellular level, using a computational model that integrates the model of intracellular calcium dynamics (Keizer and Levine 1996) and the multi-compartment neuron model (Poirazi et al. 2003).

## Methods

The calcium regulation underlying the association mechanism depends on the electrical property and structure of soma and dendrite that differ between neuron types. Regarding this point, a hetero-synaptic association has often been observed in CA1 pyramidal neurons so that we constructed a neuron model based on the multi-compartment CA1 pyramidal neuron model developed by Poirazi et al. (2003). In the following sections, we describe the details of the mathematical neuron model for our computational simulation. Our program for the simulation was coded in C and the following differential equations were solved by backward Euler method and exponential Euler method.

### Structure of computational neuron model

**Fig. 1:**
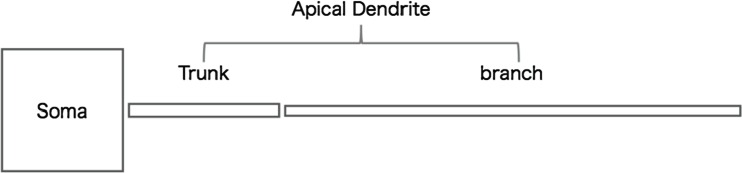
Structure of neuron model. The length of soma, dendritic trunk, and branch are 10 μm , 150 μm, and 250 μm , respectively

The neuron model consists of three cellular parts, which are trunk and branch of apical dendrite shaft and soma as shown in Fig. 1. Each cellular part is divided into cylindrical compartments where the length *L* (μm) and the radius *a* (μm) are different for the three cellular parts: for the proximal part, we set *L*_soma_ = 10 μm, *L*_trunck_ = 1 μm, *L*_branch_ = 1 μm, *a*_soma_ = 5 μm, *a*_trunk_ = 0.6 μm, and *a*_branch_ = 0.4 μm. It should be noted that spines are not explicitly included in the dendritic model.

### Electrical activity of computational neuron model

In each compartment of cellular parts, the differential equation for the membrane voltage *V* is described as

1 $$ \begin{array}{c}\kern1em {C}_{\mathrm{m}}\frac{dV}{dt}={I}_{\mathrm{Na}}+{I}_{\mathrm{K}}+{I}_{\mathrm{Ca}}+{I}_{\mathrm{h}}+{I}_{\mathrm{leak}}+{I}_{\mathrm{coup}}+{I}_{\mathrm{in}},\kern1em \\ {}\kern1em {I}_{\mathrm{K}}={I}_{\mathrm{A}}+{I}_{\mathrm{K}\mathrm{dr}}+{I}_{\mathrm{A}\mathrm{HP}},\kern1em \\ {}\kern1em {I}_{\mathrm{Ca}}={I}_{\mathrm{Ca}\mathrm{L}}+{I}_{\mathrm{Ca}\mathrm{T}}.\kern1em \end{array} $$

We set the membrane capacitance *C*_m_ to 1 μF/cm^2^.* I*_Na_ denotes voltage-dependent sodium current. *I*_K_ corresponds to the sum of potassium A current *I*_A_, delayed rectifier potassium current *I*_Kdr_, and calcium-activated potassium after-hyperpolarization current *I*_AHP_. *I*_Ca_ corresponds to the sum of high-threshold calcium current *I*_CaL_, and low-threshold calcium current *I*_CaT_. *I*_h_ denotes hyperpolarization-activated current. *I*_leak_ denotes leak current. *I*_coup_ denotes the electrical coupling between the compartments. *I*_in_ denotes current injections or synaptic currents in mA/cm2. The forms of these currents are mostly specified in Poirazi et al. (2003) and described in the following sections.

#### Voltage-dependent sodium current *I*_Na_

The model kinetics for the sodium currents *I*_Na_ is described as

2 $$ {I}_{\mathrm{Na}}={g}_{\mathrm{Na}}\kern0.1em {m}^2\kern0.1em h\kern0.1em s\kern0.1em \left({E}_{\mathrm{Na}}-V\right), $$

where *g*_Na_ is the maximal conductance of sodium currents *I*_Na_ shown in Table 1. *E*_Na_ is its reversal potential: *E*_Na_ = 50 mV. The parameters *m* and *h* are the activation and inactivation gating variables for sodium currents, respectively. An additional gating variable *s* is introduced to account for attenuation of the sodium currents (Jung et al. 1997; Migliore et al. 1999). The differential equation for the gating variables *m*, *h*, and *s* is given by

3 $$ {\tau}_X\frac{dX}{dt}={X}_{\infty }-X,\kern0.5em X=\left\{m,h,s\Big\}\right\}. $$

**Table 1:**
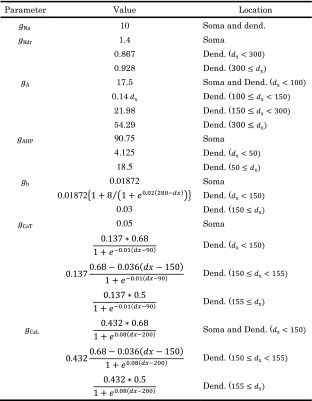
Maximum conductances depending on the cellular parts. Conductance *g* is in mS/cm^2^ and distance from soma *d*_x_ is in μm

with the following steady-state equations and time constants *τ*_X_ in ms for *m*, *h*, and *s.*

4 $$ \begin{array}{lll}{m}_{\infty }=\frac{1}{1+ \exp \left(-\frac{V+40}{3}\right)},\kern1em & {h}_{\infty }=\frac{1}{1+ \exp \left(\frac{V+45}{3}\right)},\kern1em & \\ {}{s}_{\infty }=\frac{1+N{a}_{\mathrm{att}} \exp \left(\frac{V+60}{2}\right)}{1+ \exp \left(\frac{V+60}{2}\right)},\kern1em \end{array} $$

5 $$ \begin{array}{ll}{\tau}_{\mathrm{m}}=0.05,\kern0.5em {\tau}_{\mathrm{h}}=0.5,\kern1em & {\tau}_{\mathrm{s}}=\frac{0.00333 \exp \left(0.0024\left(V+60\right)Q\right)}{1+ \exp \left(0.0012\left(V+60\right)Q\right)}.\kern1em \end{array} $$

*Na*_att_ in Eq. (4) represents the degree of sodium current attenuation and varies depending on the location parameter *d*_*x*_ (μm) as shown below, which denotes distance from soma.

6 $$ N{a}_{\mathrm{att}}=\left\{\begin{array}{cc}\kern1em 1.0\kern1em & \kern1em \mathrm{soma}\kern1em \\ {}\kern1em 0.95-0.00217dx\kern1em & \kern1em \mathrm{dendritictrunk}\left({d}_x\le 150\right)\kern1em \\ {}\kern1em 0.41\kern1em & \kern1em \mathrm{dendriticbranch}\left(150<{d}_x\le 350\right)\kern1em \\ {}\kern1em 0.65\kern1em & \kern1em \mathrm{dendriticbranch}\left(350<{d}_x\right)\kern1em \end{array}\right.. $$

*Q* in Eq. (5) is given by

7 $$ Q=\frac{F}{R\left(T+ degC\right)}, $$

where *R* is gas constant, *F* is Avogadro constant, *T* is the absolute temperature and *degC* is the temperature in degrees Celsius. In our simulation, *degC* was set to 34 °C.

#### Delayed rectifier potassium current *I*_Kdr_

The model kinetics for the delayed rectifier potassium currents *I*_Kdr_ is described as

8 $$ {I}_{\mathrm{K}\mathrm{dr}}={g}_{\mathrm{K}\mathrm{dr}}\kern0.1em {m}^2\kern0.1em \left({E}_{\mathrm{K}}-V\right), $$

where maximum conductance *g*_Kdr_ is shown in Table 1. Reversal potential of potassium currents *E*_K_ is −80 mV. The differential equation for the gating variables *m* is the same as Eq. (3). The steady-state equation *m*_∞_ and time constant *τ*_m_ are different in soma and dendrite:

9 $$ \begin{array}{l}\begin{array}{ll}{m}_{\infty}^{\mathrm{soma}}=\frac{1}{1+ \exp \left(-\frac{V+46.3}{3}\right)},\kern1em & {m}_{\infty}^{\mathrm{dend}}=\frac{1}{1+ \exp \left(-\frac{V+42}{2}\right)},\kern1em \end{array}\kern1em \\ {}\kern1em {\tau}_{\mathrm{m}}^{\mathrm{soma}}=2.2,\kern0.5em {\tau}_{\mathrm{m}}^{\mathrm{dend}}=3.5.\kern1em \end{array} $$

#### A-type potassium current I_A_

The model kinetics for the A-type potassium currents *I*_A_ is described as

10 $$ {I}_{\mathrm{A}}={g}_{\mathrm{A}}mh\left({E}_{\mathrm{K}}-V\right), $$

where maximum conductance *g*_A_ is shown in Table 1. The differential equation for the gating variables *m* and *h* is the same as Eq. (3). The steady-state equation *and* time constant for *m* are different in proximal (soma or *dx* ≤ 100) and distal (*dx* > 100) compartments:

11 $$ \begin{array}{ll}{m}_{\infty}^{\mathrm{prox}}=\frac{1}{1+ \exp \left(0.001\zeta (V)\left(V-11\right)Q\right)},\kern1em & \\ {}{\tau}_{\mathrm{m}}^{\mathrm{prox}}= \max \left(\frac{ \exp \left(0.00055\zeta (V)\left(V-11\right)Q\right)}{0.05\mathrm{qt}\left(1+ \exp \left(0.001\zeta (V)\left(V-11\right)Q\right)\right)},0.1\right),\kern1em \end{array} $$

12 $$ \begin{array}{ll}{m}_{\infty}^{\mathrm{dist}}=\frac{1}{1+ \exp \left(0.001\zeta (V)\left(V+1\right)Q\right)},\kern1em & \\ {}{\tau}_m^{\mathrm{dist}}= \max \left(\frac{ \exp \left(0.00039\zeta (V)\left(V+1\right)Q\right)}{0.1 qt\left(1+ \exp \left(0.001\zeta (V)\left(V+1\right)Q\right)\right)},0.1\right),\kern1em \end{array} $$

where *Q* is specified in Eq. (7) and *qt* = 5^(*degC* - 24)/10^. The function *ζ*(*V*) in Eq. (11) and Eq. (12) differs depending on the location of compartment as shown below.

13 $$ \begin{array}{ll}{\zeta}^{\mathrm{prox}}(V)=-1.5-\frac{1}{1+ \exp \left(\frac{V+40}{5}\right)},\kern1em & \\ {}{\zeta}^{\mathrm{dist}}(V)=-1.8-\frac{1}{1+ \exp \left(\frac{V+40}{5}\right)}.\kern1em \end{array} $$

On the other hand, the steady-state equation and time constant for *h* are uniform:

14 $$ \begin{array}{ll}{h}_{\infty }=\frac{1}{1+ \exp \left(0.003\left(V+56\right)Q\right)},\kern1em & {\tau}_{\mathrm{h}}= \max \left(0.26\left(V+50\right),2\right).\kern1em \end{array} $$

#### Calcium-dependent potassium current *I*_AHP_

The model kinetics for the calcium-dependent potassium currents *I*_AHP_ is described as

15 $$ {I}_{\mathrm{AHP}}={g}_{\mathrm{AHP}}m\left({E}_{\mathrm{K}}-V\right), $$

where maximum conductance *g*_AHP_ is shown in Table 1. The differential equation for the gating variables *m* is same as Eq. (3). The steady-state equation *m*_∞_ and time constant *τ*_m_ are given by

16 $$ \begin{array}{c}\kern4.25em {m}_{\infty }=\frac{\alpha (V)}{\alpha (V)+\beta (V)},\kern1em \\ {}\begin{array}{ll}\alpha (V)=\frac{0.48}{1+\frac{0.18}{C{a}_{\mathrm{cyt}}} \exp \left(-1.68VQ\right)},\kern1em & \beta (V)=\frac{0.28}{1+\frac{C{a}_{\mathrm{cyt}}}{0.011} \exp (2VQ)},\kern1em \end{array}\kern1em \end{array} $$

where *Ca*_cyt_ (mM) is calcium concentration in cytoplasm and *Q* is specified in Eq. (7).

#### Voltage-dependent low-threshold calcium current *I*_CaT_

The model kinetics for the low-threshold calcium currents *I*_CaT_ is described as

17 $$ \begin{array}{c}\kern1.5em {I}_{\mathrm{CaT}}={g}_{\mathrm{CaT}}{m}^2h\frac{0.001}{0.001+{C}_{\mathrm{cyt}}}GHK\left(V,{C}_{\mathrm{cyt}},{C}_{\mathrm{o}}\right),\kern1em \\ {}\kern1em GHK\left(V,{C}_{\mathrm{cyt}},{C}_{\mathrm{o}}\right)=\alpha f\left(\frac{V}{\alpha}\right)\left(1-\frac{C_{\mathrm{cyt}}}{C_{\mathrm{o}}} \exp \left(\frac{V}{\alpha}\right)\right),\kern1em \\ {}\kern1em \begin{array}{cc}\kern1em \alpha =\frac{0.085\left(T+degC\right)}{z},\kern1em & \kern1em f(x)=\frac{x}{ \exp (x)-1},\kern1em \end{array}\kern1em \end{array} $$

where maximum conductance *g*_CaT_ is shown in Table 1, *z* denotes the valence of calcium ion and *C*_o_ is the extracellular calcium concentration. *C*_o_ is set to 1 (mM). The differential equation for the gating variables *m* and *h* are same as Eq. (3). The steady-state equations and time constant for *m* and *h* are given by

18 $$ \begin{array}{ll}{m}_{\infty }=\frac{\alpha_{\mathrm{m}}(V)}{\alpha_{\mathrm{m}}(V)+{\beta}_{\mathrm{m}}(V)},\kern1em & {h}_{\infty }=\frac{\alpha_{\mathrm{h}}(V)}{\alpha_{\mathrm{h}}(V)+{\beta}_{\mathrm{h}}(V)},\kern1em \end{array} $$

19 $$ \begin{array}{ll}\ {\tau}_{\mathrm{m}}=\frac{1}{\alpha_{\mathrm{m}}(V)+{\beta}_{\mathrm{m}}(V)},\hfill &\ {\tau}_{\mathrm{h}}=\frac{1}{\alpha_{\mathrm{h}}(V)+{\beta}_{\mathrm{h}}(V)}\hfill \end{array} $$

where maximum conductance *g*_CaL_ is shown in Table 1. The differential equation for the gating variable *m* is same as Eq. (3). The steady-state equation for *m* is same as Eq. (18). *α*(*V*), *β*(*V*) and time constant for *m* are given by

22 $$ \begin{array}{c}\kern1em {\tau}_{\mathrm{m}}=\frac{0.2}{\alpha_{\mathrm{m}}(V)+{\beta}_{\mathrm{m}}(V)},\kern1em \\ {}\kern1em \begin{array}{ll}{\alpha}_{\mathrm{m}}(V)=-0.055\frac{V+27.01}{ \exp \left(-\frac{V+27.01}{3.8}\right)-1},\kern1em & {\beta}_{\mathrm{m}}(V)=0.94 \exp \left(-\frac{V+63.01}{17}\right).\kern1em \end{array}\kern1em \end{array} $$

On the other hands, kinetics equation for dendritic L-type calcium currents is given by

23 $$ {\mathrm{I}}_{\mathrm{Ca}\mathrm{L}}^{\mathrm{dend}}={g}_{\mathrm{Ca}\mathrm{L}}{m}^3h\left({E}_{\mathrm{Ca}}-V\right). $$

The differential equation for the gating variables *m* and *h* is the same as Eq. (3). The time constants are τ_m_ = 3.6 ms and τ_h_ = 29 ms and steady-state equations are given by

24 $$ \begin{array}{ll}{m}_{\infty }=\frac{1}{1+ \exp \left(-V-37\right)},\kern1em & {h}_{\infty }=\frac{1}{1+ \exp \left(\frac{V+41}{0.5}\right)},\kern1em \end{array} $$

#### Hyperpolarization activated current I_h_

The model kinetics for the hyperpolarization activated current *I*_h_ is given by

25 $$ {I}_{\mathrm{h}}={g}_h\ \mathrm{m}\ \left({E}_{\mathrm{h}}-V\right) $$

where maximum conductance ${g}_h$ is shown in Table 1. Reversal potential of currents ${E}_h$ is −10 mV. The differential equation for the gating variables *m* is same as Eq. (3). The steady-state equation ${m}_{\infty }$and time constant ${\tau}_m$ are given by

26 $$ {m}_{\infty }=\frac{1}{1+ \exp \left(-\frac{V+90}{8.5}\right)}, $$

27 $$ {\tau}_{\mathrm{m}}=\left\{\kern0.5em \begin{array}{c}\kern1em 1\kern1em \\ {}\kern1em \frac{2}{ \exp \left(-\frac{V+145}{17.5}\right)+ \exp \left(\frac{V+16.8}{16.5}\right)}+10\kern1em \end{array}\right.\kern0.5em \begin{array}{c}\kern1em V>-30\mathrm{mV}\kern1em \\ {}\kern1em \mathrm{otherwise}\kern1em \end{array}. $$

#### Leak current *I*_leak_

Stuart and Spruston suggested that membrane resistance *R*_m_ follows a sigmoidally decreasing distribution from the soma to the distal trunk of pyramidal neurons (Stuart and Spruston 1998). Therefore, membrane resistance is defined as follows in accordance with Poirazi et al. (2003):

28 $$ {R}_{\mathrm{m}}={R}_{\mathrm{m}}^{\mathrm{soma}}+\frac{R_{\mathrm{m}}^{\mathrm{end}}-{R}_{\mathrm{m}}^{\mathrm{soma}}}{1+ \exp \left(\frac{d_{\mathrm{half}}-{d}_{\mathrm{x}}}{steep}\right)}, $$

where ${R}_{\mathrm{m}}^{\mathrm{soma}}=200$ KΩcm^2^, ${R}_{\mathrm{m}}^{\mathrm{end}}=$120 KΩcm^2^, *d*_half_ = 200 μm, *steep* = 50 μm and *d*_x_ (μm) is distance from soma. The model kinetics for leak currents is described as

29 $$ {I}_{\mathrm{leak}}=\frac{1}{R_{\mathrm{m}}}\left({E}_{\mathrm{leak}}-V\right), $$

where reversal potential of leak currents *E*_leak_ = −70 mV.

#### Electrical coupling between the compartments * I*_coup_

The axial resistance *R*_a_ also decreases sigmoidally from the soma to the apical trunk:

30 $$ {R}_{\mathrm{a}}={R}_{\mathrm{a}}^{\mathrm{soma}}+\frac{R_{\mathrm{a}}^{\mathrm{end}}-{R}_{\mathrm{a}}^{\mathrm{soma}}}{1+ \exp \left(\frac{d_{\mathrm{half}}-{d}_{\mathrm{x}}}{steep}\right)}, $$

where ${R}_{\mathrm{a}}^{\mathrm{soma}}=50$ KΩcm, ${R}_{\mathrm{a}}^{\mathrm{end}}=$ 35 KΩcm, *d*_half_ = 210 μm, *steep* = 50 μm and *d*_x_ (μm) is distance from soma. The axial resistance in dendritic branch (*d*_x_ > 150) is set to the somatic value, i.e., *R*_a_ = 50 KΩcm. The model kinetics for the coupling currents of the i-th compartment is given by

31 $$ \begin{array}{l}{I}_{\mathrm{coup},\mathrm{i}}={g}_{\mathrm{i},\mathrm{i}+1}\left({V}_{\mathrm{i}+1}-{V}_{\mathrm{i}}\right)+{g}_{\mathrm{i},\mathrm{i}-1}\left({V}_{\mathrm{i}+1}-{V}_{\mathrm{i}}\right),\kern1em \\ {}{g}_{\mathrm{i},\mathrm{i}\pm 1}=\frac{a_i{a}_{i\pm 1}^2}{L_{\mathrm{i}}\left({L}_{\mathrm{i}\pm 1}{R}_{\mathrm{a},\mathrm{i}\pm 1}{a}_i^2+{L}_{\mathrm{i}}{R}_{\mathrm{a},\mathrm{i}}{a}_{\mathrm{i}\pm 1}^2\right)},\kern1em \end{array} $$

where *a*_i_ and *L*_i_ are radius and length of the i-th compartment, respectively, and these structure parameters are set to the values described in Section 2.1.

#### Synaptic current *I*_in_

*I*_in_ is the current injection in the soma or NMDAR-mediated synaptic currents from dendritic spine to the dendritic compartment. For a diffused NMDA current from the spine, we simply scaled the following double exponential model in accordance with Zador et al. (1990):

32 $$ {I}_{\mathrm{in},\mathrm{nmda}}=\alpha \frac{S_{\mathrm{s}\mathrm{p}}}{S_{\mathrm{s}\mathrm{h}}}{g}_{\mathrm{nmda}}\frac{ \exp \left(\frac{t_{\mathrm{s},\mathrm{p}\mathrm{r}\mathrm{e}}-t}{\tau_1}\right)- \exp \left(\frac{t_{\mathrm{s},\mathrm{p}\mathrm{r}\mathrm{e}}-t}{\tau_2}\right)}{1+0.00033\left[{\mathrm{Mg}}^{2+}\right] \exp \left(-0.06V\right)}\left({E}_{\mathrm{nmda}}-V\right), $$

where *g*_nmda_ is maximum conductance of NMDA, *t*_s , pre_ is spike-time of postsynaptic neuron, *τ*_1_= 80 ms, *τ*_1_= 0.67 ms, [Mg^2+^] = 1.2 mM and *E*_nmda_ = 0 mV. *S*_sp_ and *S*_sh_ are surface area of the spine head and dendritic compartment, respectively: *S*_sp_ is set to 0.1 μm^2^ in accordance with Harris and Stevens (1989). *α* is the current diffusion rate on the basis of the physiological evidence that 1 % of calcium ions flowing into a spine diffuse to the dendritic shaft (Sabatini et al. 2002): *α* is set to 0.01. *V* is the actual membrane voltage of the spine, but we consider *V* as the membrane voltage of the dendritic shaft for simplification.

### Intracellular calcium dynamics

The assumed intracellular calcium signaling is regulated by the interaction between NMDAR, VDCC, and RyR. In order to implement RyR-mediated calcium flux in the neuron model, we modified the calcium dynamics of Poirazi et al. (2003) with parameters proposed by Keizer and Levine (1996). In each compartment, the behavior of intracellular calcium ions is described as

33 $$ \frac{d{C}_{\mathrm{tot}}}{dt}={J}_{\mathrm{in}}-{J}_{\mathrm{out}}+{J}_{\mathrm{diff}}+{J}_{\mathrm{soc}}, $$

34 $$ \frac{d{C}_{\mathrm{cyt}}}{dt}={J}_{\mathrm{in}}-{J}_{\mathrm{out}}+{J}_{\mathrm{diff}}+{J}_{\mathrm{er}}, $$

35 $$ {C}_{\mathrm{er}}=\frac{1}{r}\left({C}_{\mathrm{tot}}-{C}_{\mathrm{cyt}}\right), $$

where *C*_cyt_ and *C*_er_ denote calcium concentration in the cytoplasm and ER, respectively. *C*_tot_ denotes total free calcium concentration in μM. *r* denotes effective volume ratio ER to cytoplasm, and we set *r* = 0.02 in accordance with Keizer and Levine (1996). *J* denotes calcium flux in μM/ms; these calcium fluxes are defined in the following sections.

#### Calcium flux between intra- and extracellular space

In Eq. (33) and Eq. (34), *J*_in_, *J*_out,_ and *J*_soc_ denote calcium flux between intra- and extracellular space. In particular, *J*_in_ denotes the sum of calcium influxes through NMDAR and VDCC, which is proportional to ion currents:

36 $$ {J}_{\mathrm{in}}=\frac{S}{zFVol}\left({I}_{\mathrm{Ca}}+0.1{I}_{\mathrm{in},\mathrm{nmda}}\right), $$

where *S* and *Vol* denote surface area and volume of the compartment, respectively. *J*_out_ denotes calcium efflux through plasma membrane calcium ATPase- (PMCA-) type pump:

37 $$ {J}_{\mathrm{o}\mathrm{ut}}={v}_{\mathrm{o}}\frac{C_{\mathrm{cyt}}^2}{C_{\mathrm{cyt}}^2+{K}_{\mathrm{o}}^2}, $$

where *v*_o_ = 0.024 μM/ms and *K*_o_ = 0.6 μM. *J*_soc_ denotes calcium influx through store-operated channels (SOCs) in the cell membrane, which has the role of refilling the ER with calcium ions and adjusting calcium concentration (Parekh and Putney 2005). The detail of calcium-regulation via SOC has not been revealed, but it was observed that SOC-mediated calcium refilling occurs when calcium stores in the ER become depleted. Therefore, we define *J*_soc_ as

38 $$ {J}_{\mathrm{s}\mathrm{oc}}={v}_{\mathrm{s}}\frac{K_{\mathrm{s}}}{C_{\mathrm{cyt}}+{K}_{\mathrm{s}}}, $$

where *v*_s_ = 0.4 μM/ms and *K*_s_ = 0.1μM.

#### Intracellular calcium diffusion

In Eq. (33) and Eq. (34), *J*_diff_ denotes intracellular calcium flux between the compartments. The intracellular calcium flux in the i-th compartment due to diffusion is described as

39 $$ \begin{array}{l}{J}_{\mathrm{diff},\mathrm{i}}=\frac{D}{\pi {a}_{\mathrm{i}}^2{L}_{\mathrm{i}}}\left\{{\delta}_{\mathrm{i},\mathrm{i}+1}\left({C}_{\mathrm{cyt},\mathrm{i}+1}-{C}_{\mathrm{cyt},\mathrm{i}}\right)+{\delta}_{\mathrm{i},\mathrm{i}-1}\left({C}_{\mathrm{cyt},\mathrm{i}-1}-{C}_{\mathrm{cyt},\mathrm{i}}\right)\right\},\kern1em \\ {}\kern1em {\delta}_{\mathrm{i},\mathrm{i}\pm 1}=2\left\{\frac{\pi {a}_{\mathrm{i}}^2{L}_{\mathrm{i}}+\pi {a}_{\mathrm{i}\pm 1}^2{L}_{\mathrm{i}\pm 1}}{{\left({L}_{\mathrm{i}}+{L}_{\mathrm{i}\pm 1}\right)}^2}\right\},\kern1em \end{array} $$

where *C*_cyt , i_ denotes the calcium concentration in the i-th compartment. *a*_i_ and *L*_i_ are the radius and length of the i-th compartment, respectively, and these structural parameters are set to the values described in Section 2.1. *D* denotes the diffusion coefficient of calcium ion and we initiated *D* = 13 *μ*m^2^/s in accordance with Allbritton et al. (1992). The value of the diffusion coefficient implicitly takes the effect of calcium buffering into account.

#### Intracellular calcium flux between ER and cytoplasm

In Eq. (33) and Eq. (34), *J*_er_ denotes intracellular calcium flux between the ER and cytoplasm, which is described as

40 $$ {J}_{\mathrm{er}}=\left({v}_{\mathrm{rel}}{P}_{\mathrm{open}}+{v}_{\mathrm{leak}}\right)\left({C}_{\mathrm{er}}-{C}_{\mathrm{cyt}}\right)-{v}_{\mathrm{p}\mathrm{ump}}\frac{C_{\mathrm{cyt}}^2}{C_{\mathrm{cyt}}^2+{K}_{\mathrm{p}}^2}. $$

On the right side of Eq. (40), the first term represents calcium release through RyR and passive calcium leak from the ER to cytoplasm. The second term represents calcium release from the cytoplasm to ER through sarco/endoplasmic reticulum calcium ATPase- (SERCA-) type pump. The behavior of ER calcium regulation depends on parameter *v*; we initiated *v*_rel_ = 0.005ms^−1^, *v*_leak_ = 0.00012 ms^−1^, and *v*_pump_= 0.12 μM/ms. *P*_open_ denotes the open probability of RyR, which depends on calcium concentration in cytoplasm. The temporal development of *P*_open_ is described with the relaxation equation of *w*:

41 $$ {P}_{\mathrm{open}}=\frac{w\left(1+{C}_{\mathrm{cyt}}^3/{K}_{\mathrm{b}}\right)}{K_{\mathrm{a}}/{C}_{\mathrm{cyt}}^4+1+{C}_{\mathrm{cyt}}^3/{K}_{\mathrm{b}}}, $$

42 $$ \tau \frac{dw}{dt}={w}_{\infty }-w, $$

43 $$ \begin{array}{ll}\kern0.5em {w}_{\infty }=\frac{K_{\mathrm{a}}/{C}_{\mathrm{cyt}}^4+1+{C}_{\mathrm{cyt}}^3/{K}_{\mathrm{b}}}{1/{K}_c+{K}_{\mathrm{a}}/{C}_{\mathrm{cyt}}^4+1+{C}_{\mathrm{cyt}}^3/{K}_{\mathrm{b}}},\kern1em & \tau ={w}_{\infty }/{K}_{\mathrm{d}},\kern1em \end{array} $$

where the kinetics parameter *K* was specified in Keizer and Levine (1996): *K*_a_ = 0.0192 μM^4^, *K*_b_ = 0.2573μM^3^, *K*_c_= 0.0571 and *K*_d_= 0.0001 ms^−1^.

## Results

**Fig. 2:**
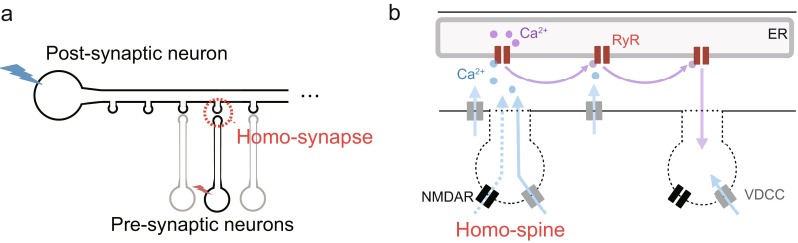
Model of CA1 pyramidal neuron and intracellular calcium signaling. **a** Schematic figure for the protocol to induce calcium fluxes by a presynaptic input and a postsynaptic action potential. **b** Schematic figure for calcium signaling activated by spike-triggered calcium influx via NMDAR and VDCC and by calcium release from the ER via RyR. The spatial distribution of RyR in the dendrite shaft was assumed to be uniform; the constant value of calcium releasing parameter in Eq. (40) represented a uniform density of RyRs. The value of calcium pumping and leaking parameters in Eq. (40) were similar to the releasing parameter. The calcium signaling started at the dendritic compartment of the target synapse (*red circle* in (**a**)), which was evoked by paired firing of the pre- and postsynaptic neurons. When the postsynaptic neuron fired, VDCC-mediated calcium influx (*solid blue arrows* in (**b**)) occurred within the range of the back-propagating action potential. On the other hand, when the presynaptic neuron fired, NMDAR-mediated calcium influx (*dashed blue arrow* in (**b**)) locally occurred at the target spine, and a portion of the calcium ions diffused to the dendrite compartment. It should be noted that spines were not explicitly incorporated into our model, but the diffused calcium flux from a spine to the dendritic shaft was modeled. The spike-evoked calcium influxes via NMDAR and VDCC activated RyR and induced the first calcium release from ER. Subsequently, the released calcium ions activated neighboring RyRs and calcium ions propagated in the dendritic shaft (*purple arrow* in (**b**))

A previous physiological study suggested that store-regulated calcium signaling in dendrites underlies the mechanism of hetero-synaptic association (Nishiyama et al. 2000). However, it is not known how calcium store (ER) regulates calcium signaling in the dendritic shaft. Figure 2 describes hypothesized calcium signaling in the situation where hetero-synaptic association occurs. Calcium signaling consists of two significant processes that are mainly regulated by RyR on ER. The first process is RyR-mediated calcium release from the ER induced by spike-triggered calcium influx via NMDAR and VDCC. The second process is the propagation of RyR activation; calcium ions released from the ER diffuse along cytoplasm and then activate RyRs at neighboring sites. Therefore, by using the described computational model, we simulated spatiotemporal calcium dynamics along the following protocols focusing on the two calcium-signaling processes and investigated the potential role of RyR-calcium regulation.

### The fundamental behavior of the computational model

**Fig. 3:**
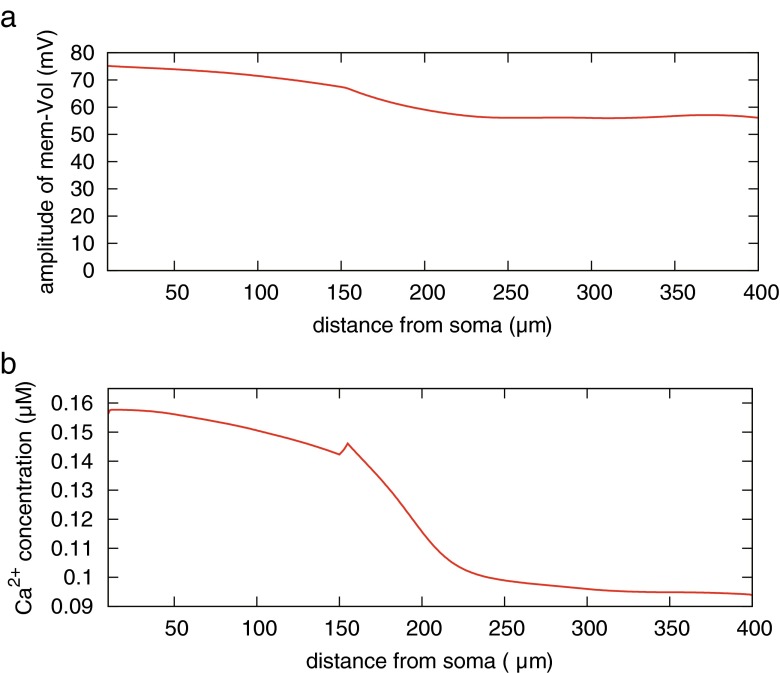
Amplitude profiles of back-propagating action potential (b-AP) and calcium transients. **a** Change in the amplitude of b-AP along a dendrite. **b** Peak of calcium transient induced by b-AP

Before performing the model simulation, we confirmed the fundamental behavior of our model. We observed the change in amplitude of dendritic membrane potential when b-AP was generated all at once from the soma (Fig. 3(a)). In this test, b-AP is generated by stimulating the soma with an external input current *I*_in_ = 0.05 mA/cm^2^ for 5 ms. As shown in Fig. 3(a), the peak amplitude of the dendritic b-AP decreases along the dendrite. The simulation result reproduces experimental results measured by patch clamp method in CA1 pyramidal neurons (Golding et al. 2001). Subsequent to b-AP, the membrane potential changed toward depolarization, and calcium influx to cytoplasm through VDCCs occurred in each dendritic compartment as shown in Fig. 3(b). The compartment at a distance of 150 μm away from soma is the dendritic junction of trunk and branch. Therefore, 150 μm away from soma, the level of calcium increase was significantly attenuated. The simulation result qualitatively matches the experimental result of Gasparini et al. (2007).

### The spike-timing dependence of RyR-mediated calcium release

We confirmed the reaction of RyR to the temporal pattern of the two calcium influxes evoked by a pair of pre- and postsynaptic spikes. In the simulation, we observed the behavior of RyR at a local site in a postsynaptic dendrite that was 200 μm away from the soma and received pre-synaptic input. The spike timing of the presynaptic neuron was controlled by tuning *t*_s_ in Eq. (32). NMDAR-mediated calcium influx only occurred at the induction site. The conductance of NMDAR-mediated synaptic current *g*_n_ in Eq. (32) was 0.118 mS/cm^2^. The spike of postsynaptic neuron was generated and propagated along the dendrite by giving an external input current *I*_in_ = 0.05 mA/cm^2^ to soma; the b-AP induces VDCC-mediated calcium influx as shown in Fig. 3(b).

**Fig. 4:**
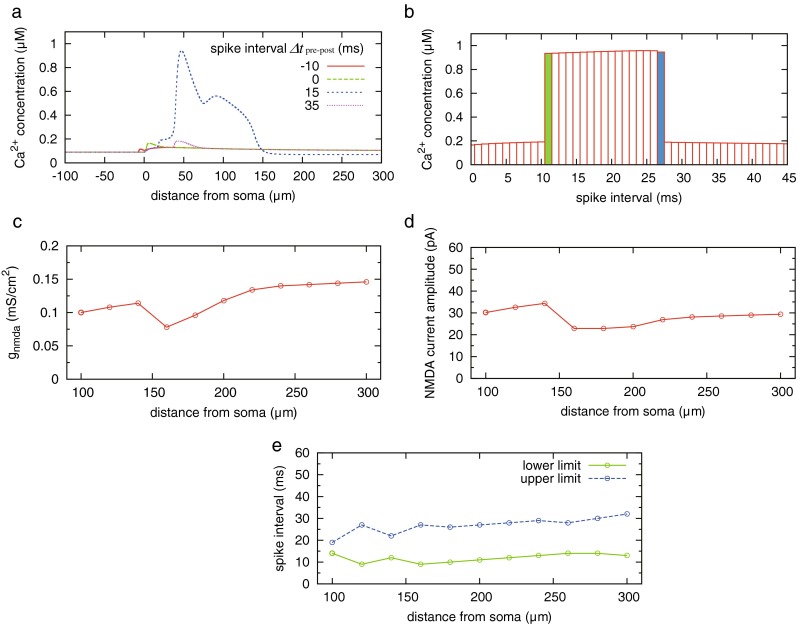
Dependence of calcium release on timings of pre- and postsynaptic spikes. **a** Calcium increase relies on the order of pre-and postsynaptic spikes. *Δt*_pre-post_ denotes spike-time interval between pre- and postsynaptic neurons. The colored lines denote the change of cytoplasmic calcium concentration at induction site 200 μm away from the soma. **b** Spike timing dependent calcium increase. ∆*t*_pre - post_ denotes the pre-to-post synaptic inter-spike intervals. The green and blue boxes are lower and upper limit of the spike-time window. (**c**, **d**, **e**) Difference of spike-time window for inducing calcium-release in each dendritic location. **c** Red circle denotes the minimum NMDA-conductance in mS/cm^2^ to induce calcium release from the ER at each site. **d** The estimated amplitude of NMDA-current observed in a spine. The current is simulated under the condition that membrane voltage is clamped to −80 mV and [Mg^+^] is set to 0 mM in accordance with physiological experiments (Andrásfalvy and Magee 2001). **e** The green and blue circles denote lower and upper limit of spike-time window in which calcium release could be induced

First, we controlled the order of pre- and postsynaptic spikes. In the simulation, the spike-time of the presynaptic neuron was fixed at 0 ms, and we adjusted the spike-time of the postsynaptic neuron. Figure 4(a) shows the change of cytoplasmic calcium concentration in the cases where the postsynaptic spike occurred at −10, 0, 15, and 35 ms. It was observed that RyR-regulated calcium release depends on the order of the spikes. Supralinear calcium increase occurred when the presynaptic spike-triggered calcium influx via NMDAR preceded the postsynaptic spike-triggered calcium influx via VDCC. In addition to the above result, we confirmed the time-window in which calcium-release was induced by paired calcium influxes. As shown in Fig. 4(b), the supralinear calcium increase occurred when the presynaptic led the postsynaptic spike by 11 to 27 ms. It showed that RyRs were activated and released calcium from the ER under the specific condition of synchronization of spike-triggered calcium influxes.

The above results presented in Fig. 4(a, b) were observed in the case where the induction site of RyR-mediated calcium release was fixed at 200 μm away from the soma. Subsequently, we changed the location of the induction site and confirmed the difference in the spike-timing dependence of calcium release. For this simulation, we set the conductance *g*_n_ in Eq. (32) to the value that was necessary to induce calcium release at the induction site. Consequently, NMDA-conductance per surface area (red circles) was almost constant but slightly increased along the dendrite (Fig. 4(c)). In contrast, the predicted amplitude of synaptic current via NMDAR in the spine (Fig. 4(d)) was constant at any location except around the dendritic junction. Under this condition, the spike-time window became longer and shifted slightly upwards along the dendrite (Fig. 4(e)).

### The generation mechanism of supralinear calcium increase

Fig. 4 shows that the bifurcation occurring in calcium dynamics depended on the temporal order and interval of calcium influxes. In this section, we investigate the generation mechanism of the bifurcation using phase plane analysis. The bifurcation phenomenon was generated at a local site in the dendrite. Therefore, we analyzed local calcium dynamics by approximating and reducing the model equations described in Section 2.3. We assumed that intracellular calcium flux between neighboring compartments does not occur: *J*_diff_ = 0 in Eq. (33) and Eq. (34). Furthermore, the open probability of RyR (*P*_open_) relaxes into steady state instantly: *w* = *w*_∞_ in Eq. (41). Under this condition, setting the right sides of Eq. (33) and Eq. (34) equal to zero derives the following nullclines:

44 $$ {C}_{\mathrm{tot}}={C}_{\mathrm{cyt}}+r{K}_{\mathrm{s}}\left(\frac{v_{\mathrm{s}}}{J_{\mathrm{out}}-{J}_{\mathrm{in}}}-1\right), $$

45 $$ {C}_{\mathrm{tot}}=\left(1+r\right){C}_{\mathrm{cyt}}+r\frac{J_{\mathrm{out}}-{J}_{\mathrm{in}}+{v}_{\mathrm{p}\mathrm{ump}}\frac{C_{\mathrm{cyt}}^2}{C_{\mathrm{cyt}}^2+{K}_{\mathrm{p}}^2}}{v_{\mathrm{rel}}{P}_{\mathrm{open}}^{\infty }+{v}_{\mathrm{leak}}}. $$

**Fig. 5:**
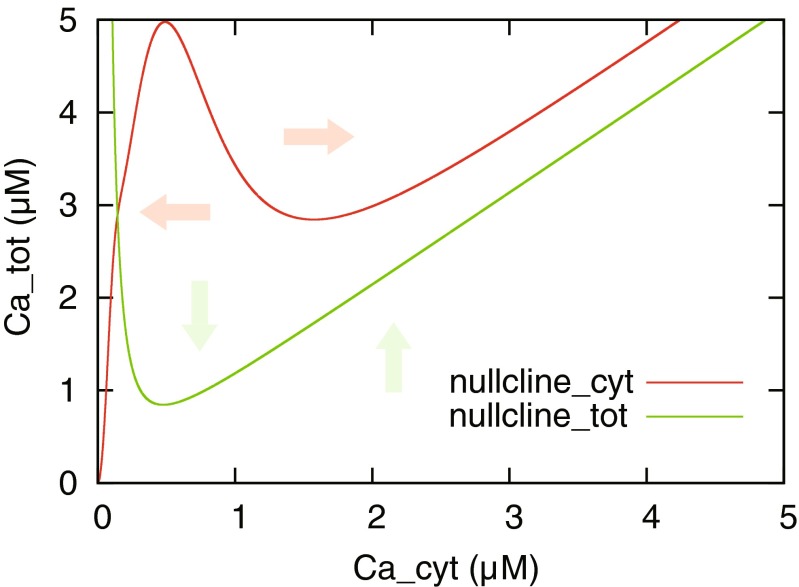
Nullclines in phase plane consisting of *C*_tot_ and *C*_cyt_. The green and red lines denote the nullclines described in Eq. (44) and Eq. (45), respectively. The green and red arrows indicate directions of changes of *C*_cyt_ and *C*_tot_ in the areas divided by the nullclines, respectively

Figure 5 shows the phase plane that is the state space of *C*_tot_ and *C*_cyt_. In the figure, the nullclines of Eq. (44) and Eq. (45) are described as green and red lines, respectively. By observing these nullclines in the phase plane, we understand the qualitative behavior of the model. For instance, when below the *C*_tot_-nullcline (green line), the state of the model tends to shift upward; this means that calcium concentration in the ER tends to increase. In addition, when above the *C*cyt-nullcline (red line), the state of the model tends to shift to the right: this means that calcium concentration in cytoplasm tends to increase. In particular, since the *C*_cyt_-nullcline indicates an increase or decrease of cytoplasmic calcium concentration (*C*_cyt_) it is considered the boundary representing whether RyR-mediated supralinear calcium increase occurs in cytoplasm.

Based on these observations, we confirmed the temporal behavior of the model and the nullclines to paired calcium influxes via NMDAR and VDCC. We then re-defined the equation of calcium influx (*J*_in_) with the simplification of the calcium dynamics model, which is described in the following simple equation:

46 $$ \begin{array}{c}\kern1em {J}_{\mathrm{in}}={J}_{\mathrm{vdcc}}+{J}_{\mathrm{nmda}},\kern1em \\ {}\kern1em {J}_{\mathrm{X}}={\alpha}_{\mathrm{X}}\left( \exp \left(\frac{t_{\mathrm{X}}-t}{\tau_{1,\mathrm{X}}}\right)- \exp \left(\frac{t_{\mathrm{X}}-t}{\tau_{2,\mathrm{X}}}\right)\right),\kern1em \end{array} $$

where *J*_in_ denotes the sum of the calcium influxes through NMDAR and VDCC. The two calcium influxes are about the same as described in Eq. (32) with different parameters: *α*_nmda_ = 8.17, *τ*_1 , nmda_ = 80 ms, *τ*_2 , nmda_ = 0.67ms, *α*_vdcc_ = 0.041, *τ*_1 , vdcc_ = 0.1 ms, and *τ*_2 , vdcc_ = 0.05 ms. In Eq. (46), *t*_X_ is the time when NMDAR- or VDCC-mediated calcium influx occurs.

**Fig. 6:**
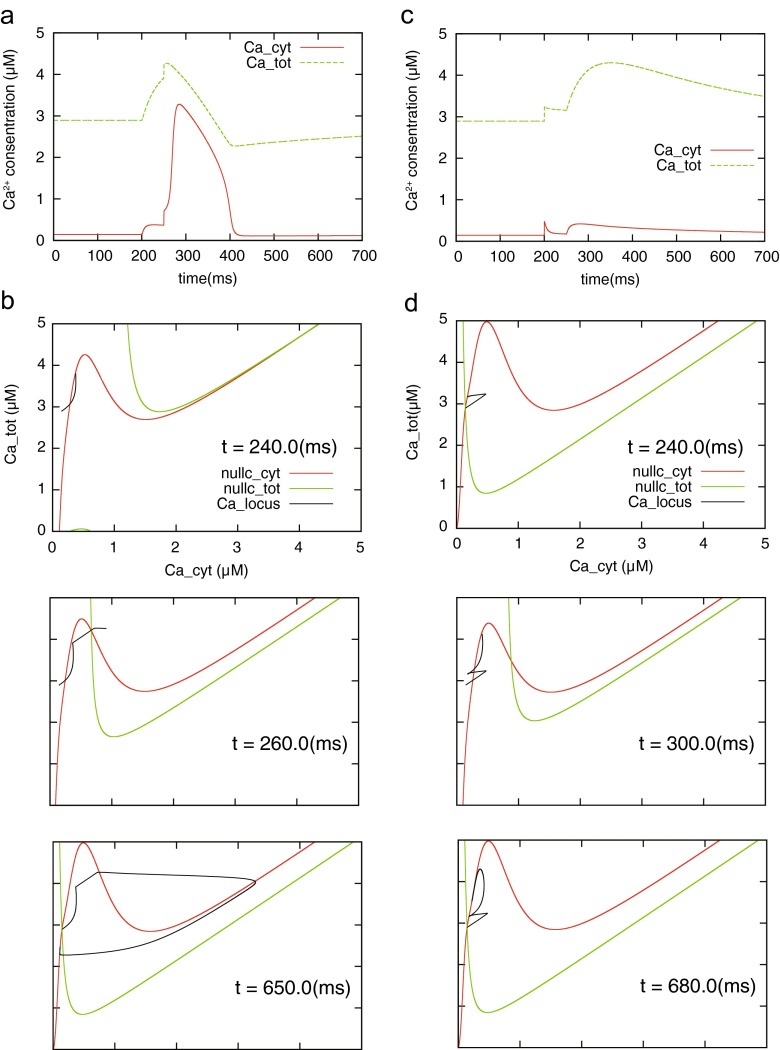
Difference in model behaviors in the order of spike-evoked calcium influxes. The temporal change of calcium concentration up to a specific time shown in (**a**) and (**c**) are described as black lines in (**b**) and (**d**) with nullclines, respectively. **a** Occurrence of calcium release. **b** Trajectory of the state before the second spike (*top*), after the second spike (*middle*), and in the steady state (*bottom*). **c** Failure of calcium release. **d** Same as (**b**) but illustrating the trajectories in the case of failure of calcium release shown in (**c**)

First of all, in Fig. 6(a) we show the time series of the model’s state under the condition that an NMDAR-mediated calcium influx precedes a VDCC-mediated influx: *t*_nmda_ = 200 ms and *t*_vdcc_ = 250 ms. Figure 6(b) shows the temporal locus of the model’s state and nullclines at 240, 260, and 650 ms in the phase plane. As shown in Fig. 6(b) top, the locus of the model’s state shifts upward in the phase plane with the occurrence of NMDAR-mediated calcium influx; this means that fluxed calcium ions are immediately pumped up by the SERCA-type pump and stored in ER because NMDAR-mediated calcium influx is slow. Subsequently, the model solution rises but does not go over the *C*_cyt_-nullcline, so that calcium release from ER does not occur. However, the following VDCC-mediated calcium influx causes the model solution to pass through the *C*_cyt_-nullcline (Fig. 6(b) center) and trigger the supralinear cytoplasmic calcium increase mediated by RyR (Fig. 6(b) bottom). In distinction to the above, Fig. 6(c) shows the results for the case where a VDCC-mediated calcium influx precedes an NMDAR-mediated influx: *t*_nmda_ = 250 ms and *t*_vdcc_ = 200 ms. The preceding calcium influx through VDCC is fast and increases cytoplasmic calcium concentration momentarily, so that the model solution transferred rightward in the phase plane (Fig. 6(d) top). However, this influx cannot induce calcium release from the ER at that time because the ER does not contain sufficient calcium ions. Subsequently, due to the following NMDAR-mediated calcium influx, ER is filled with calcium ions but the calcium release from the ER is not induced (Fig. 6(d) center, bottom). This is because the following cytoplasmic calcium increase is insufficient to activate RyR on ER due to the SERCA-type calcium pump activity. According to the phase plane analysis for the local calcium dynamics model, we confirmed that RyR-mediated calcium release occurred when the calcium concentration in cytoplasm sufficiently increased under the condition of a high calcium concentration in the ER. NMDAR-mediated calcium influx was slow and persistent, consequently being responsible for maintaining a high calcium concentration in the ER. In contrast, a VDCC-mediated calcium influx was rapid and transient, so that it contributed to a rapid increase in calcium concentration in cytoplasm. Taken together, it was found that supralinear calcium increase tended to occur as shown in Fig. 4, when an NMDAR-mediated calcium influx preceded a VDCC-mediated influx, that is, a presynaptic spike preceded a postsynaptic spike.

### **Calcium propagation based on chain activation of RyRs**

**Fig. 7:**
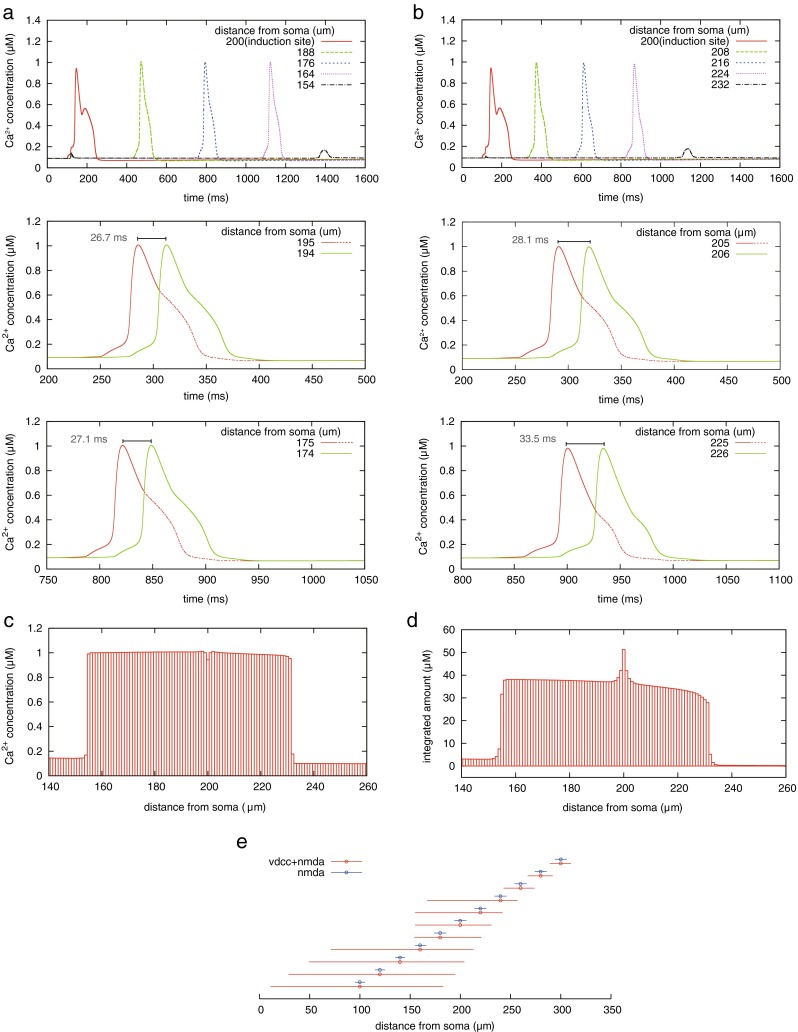
Intracellular calcium propagation based on chain activation of RyRs. (**a**, **b**) Propagation of calcium-increase in the dendrite, (**a**) and (**b**) showing calcium propagation toward soma and toward end of the dendrite, respectively. Top. Colored lines denote the change of calcium concentration at different sites of the dendrite. Center. Calcium increases at 5 (*red*) and 6 (*green*) μm away from the induction site toward soma (**a**) and toward end of the dendrite (**b**). Bottom. Same as for center but for distances of 25 and 26 μm away from induction site toward soma and dendrite end. As the chain of calcium release propagates from the induction site, the time interval of spike-like calcium increase becomes longer and the shape of calcium increase tapers. (**c**) Peak amount of calcium increase in the calcium propagation shown in (**a**) and (**b**). The propagation suddenly stops at a certain site. (**d**) Level of calcium increase at each site of the dendrite. The colored bars denote the integrated amount of increased calcium concentration that is surplus above a certain threshold. (**e**) The range of calcium propagation in the dendrite. The circles represent an induction site of calcium propagation and the length of the horizontal bars represents the sites where calcium release occurred. Red represents calcium propagation induced by paired spikes of pre- and postsynaptic neurons. Blue represents the case where the postsynaptic spike does not occur, and calcium release is only induced by presynaptic spike

After the paired spikes evoked a calcium increase at the local dendritic site, neighboring RyRs were activated, and calcium release from the ER was propagated in the dendrite. Figure 7(a, b) shows the change of cytoplasmic calcium concentration at each site of the dendrite, when supralinear calcium increase occurred 200 μm away from the soma, as shown in Fig. 4(a). The spike-like calcium-increase propagated bidirectionally along the dendrite and its speed depended on its direction. In the calcium propagation, the peak amount of calcium increase was mostly constant and the propagation suddenly stopped at a certain point (Fig. 7(c)); this is because the degree of RyR-regulated calcium release was all-or-none. In addition, the total amount of increased calcium concentration was maximal at the induction site of the calcium propagation. However, within the propagation range, except for the induction site, there was no difference in the integrated amount of increased calcium concentration (Fig. 7(d))

Although the propagation distance was about 30–50 μm shown in Fig. 7(a, b), it differed depending on the location where the first calcium release was inducted by the paired calcium influxes through NMDAR and VDCC (Fig. 7(e)). As the induction site of propagation moved closer to the soma, the change of calcium concentration propagated more widely. In the case where the induction site was closer to the dendritic junction and at 150 μm away from soma, the propagation range centering on the starting point was asymmetric. This is because the spatial distribution of VDCC-expression was not homogeneous along the dendrite, as shown in Fig. 3(b). Furthermore, in the case where the induction site was 180, 200, 220 μm away from the soma, the propagation toward the soma stopped around the dendritic junction.

### Effects of model parameters on calcium dynamics

The behavior of RyR calcium regulation depends on the three model *v* parameters in Eq. (40). Therefore, we examined the effect of these parameters on RyR-regulated calcium flux in cytoplasm.

**Fig. 8:**
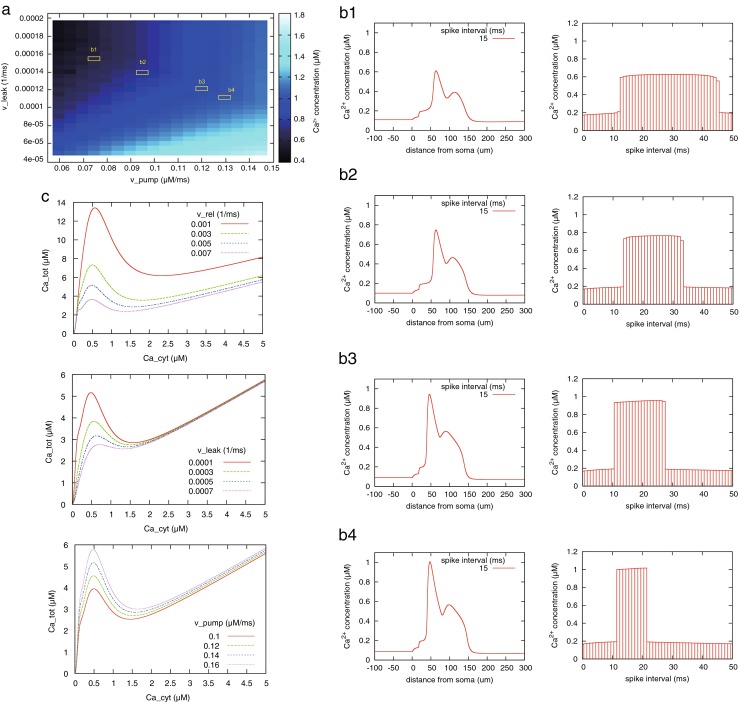
Relation between model parameters of the ER and calcium dynamics at the induction site. *v*_rel_ and *D* are fixed to 0.005 ms^−1^ and 0.013 μm^2^/ms, respectively. The location of the induction site is 200 μm away from the soma. Paired spikes of pre- and postsynaptic neurons induce calcium release; the spike interval is 15 ms. **a** Color brightness corresponds to the peak amount of calcium increase at the induction site. **b** Time course of cytoplasmic calcium concentration (*left*) and dependence of calcium increase on the spike interval (*right*) using the parameter set surrounded by yellow line in (**a**). **c** Relation between one model parameter and *C*_cyt_-nullcline. *C*_cyt_-nullcline implies the threshold for calcium release: when time locus of *C*_cyt_ and *C*_tot_ surpasses the nullcline, the model behavior is bifurcated and calcium-release occurs

First, we observed the relation between model parameters and peak amount of calcium increase at the induction site 200 μm away from the soma (Fig. 8(a, b)). In Fig. 8(a), as the degree of calcium pumping is higher and calcium leak is lower, the peak amount of calcium concentration increased from 0.4 μM to 1.7 μM. This simulation result can be accounted by the dependence of nullcline of the model on the model parameters (Fig. 8(c)). For instance, as the degree of calcium pumping (*v*_pump_) increases, the local maximum point of *C*_cyt_-nullcline shifts up in the phase plane. In this case, the time locus of the equilibrium point cannot surpass the nullcline and more calcium influx is required. In other words, cytoplasmic calcium concentration is difficult to increase and calcium release tends not to be triggered due to the high degree of calcium pumping. Meanwhile, more calcium ions are stored in the ER; therefore, the degree of calcium release is increased as *v*_pump_ increases.

**Fig. 9:**
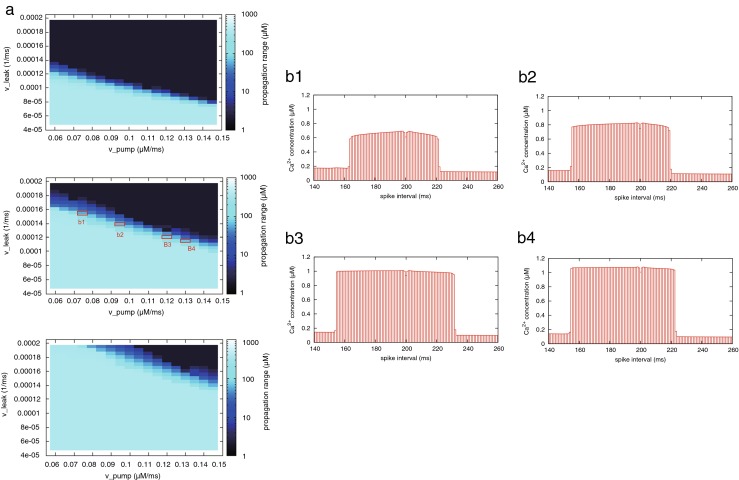
Relation between model parameters of the ER and the range of calcium propagation. **a** Color brightness corresponds to the total distance of bidirectional calcium propagation from the induction site. Top, center, and bottom figures show the difference in the case where *v*_rel_ = 0.004, 0.005, 0.006 ms^−1^, respectively. **b** Peak amount of calcium increase in each site of the dendrite using the parameter set surrounded by red line in (**a**)

Next, we examined the effect of the model parameters on calcium propagation (Fig. 9(a)). In the color maps, spatial change of brightness is discrete. It means that the parameter set is mostly categorized into two regions: one represents propagation that does not occur, and the other represents propagation that does not stop. In addition, the range of calcium propagation critically depends on the model parameters. For instance, in the case where *v*_leak_ and *v*_pump_ are fixed to 0.00012 ms^−1^ and 0.12 μM/ms, respectively, and *v*_rel_ is changed from 0.004 to 0.006 ms^−1^, the range of calcium propagation is highly dependent on *v*_rel_. Figure 9(b) shows that although the peak amount of propagated calcium increase also depends on the parameters, propagated calcium increase does not tend to attenuate.

## Discussion

As a result of the simulation, we observed that paired spikes of pre- and postsynaptic neurons induced RyR-regulated calcium release at the induction site, and the released calcium ions initiated intracellular calcium propagation based on the chain activation of RyRs. In this section, based on the physiological and theoretical knowledge of calcium-dependent synaptic plasticity, we discuss the role and effect of RyR-regulated calcium dynamics on collective synaptic change.

### Dependence of calcium release on spike timing of neurons

Because it has been suggested that synaptic plasticity underlies formation and maintenance of the functional neural circuit, the mechanism of activity-dependent synaptic plasticity has been intensively studied. In particular, the experimental study by Bi and Poo showed the temporal aspect of synaptic plasticity; whether synaptic efficacy is potentiated or depressed depends on spike timings of pre- and postsynaptic neurons (Bi and Poo 1998). In general, pre-to-post and post-to-pre spike timings induce long-term potentiation and depression, respectively, and the amount of change in synaptic efficacy depends on the spike intervals. Furthermore, it has been observed that spike-timing-dependent synaptic change at one synapse (homo) induces synaptic change at other synapses (hetero), and the changes in hetero-synapses also depend on the spike timings at the homo-synapse (Nishiyama et al. 2000). However, the detailed mechanism of such a spatial association of synaptic plasticity is still unclear. The spatial association of synaptic plasticity is presumably based on calcium propagation from homo-synapse to hetero-synapses along dendrites. In addition, it is suggested that calcium store (ER) mainly regulates the dendritic calcium dynamics to control cytoplasmic calcium concentration by storing and releasing calcium ions. Although the calcium release might be regulated by the complex interaction of IP_3_R and RyR on ER, we focused on the unidentified function of RyR-calcium regulation in dendritic calcium signaling, which underlies the hetero-synaptic association.

Assuming that the spatial association of synaptic plasticity between synapses is mainly mediated by the dendritic calcium dynamics, calcium store with RyR could play a role for linking the temporal activity of pre- and postsynaptic neurons with synaptic plasticity. Regarding this point, our simulation results showed that the paired calcium influxes evoked by paired spikes in pre-to-post order could induce calcium release via RyR from the ER (Fig. 4). The induction process of calcium release consisted of the following two steps: the first step was filling up the ER with calcium ions, and the second step was increasing cytoplasmic calcium concentration sufficiently to cause threshold-like behavior through the bifurcation as shown in Fig. 6. NMDAR and VDCC contributed differently to these steps; that is, a slow but persistent calcium influx via NMDAR was responsible for the first step and contributed to bottom-up cytoplasmic calcium concentration over the long term. However, this mechanism was not responsible for the second step because its low amplitude of calcium increase was insufficient to trigger calcium release. On the other hand, transient but fast calcium influx via VDCC caused a high calcium increase in the cytoplasm as the second step but was not suitable for the first step because its duration was too short for calcium ions to be pumped into the ER. If the calcium pumping rates and RyR in Eq. (40) are increased, VDCC could fill the ER with calcium ions and NMDAR could trigger calcium release. However, in this case, the segregation of the contributions is lost; either one of the two could fill up the ER and trigger calcium release by itself. From the processes described above, calcium release tended to occur when a presynaptic spike preceded a postsynaptic one.

### Dependence of the time-window on distance from the induction site

Figure 4(c, d) suggests that the expression level of NMDAR was almost constant but slightly increased along the dendrite shaft except around the dendritic junction, and the predicted peak amplitude of NMDAR-current was in accordance with a physiological study focusing on the location dependence of density of glutamate receptors (Pettit and Augustine 2000; Andrásfalvy and Magee 2001). The almost uniform distribution of NMDAR can be attributed to the fact that the peak amount of calcium influx via VDCC attenuates along the dendrite (Fig. 3(b)). At the distal site, where the amount of VDCC-mediated calcium influx is low, cytoplasmic calcium concentration is required to be largely increased by NMDAR-mediated calcium influx so as to induce calcium release from the ER, as the phase plane analysis suggested (Fig. 6(b) and (d)). Even if the conductance density of NMDAR-mediated current is uniform, the increase in cytoplasmic calcium concentration brought about by NMDAR-mediated current is larger at the thinner dendrite because the constant of the conversion from current density to concentration was a function of the inverse of the dendrite’s diameter (Eq. (36)). Therefore, the almost uniform conductance density of NMDAR-mediated current along the dendrite could generate a sufficient increase in cytoplasmic calcium concentration to induce calcium release even at a distal site. In general, as the distance of a synapse from the soma increases, it takes more time for a presynaptic input from the synapse to arrive at the soma, and it also takes longer for the generated action potential to propagate back to the synapse. Namely, the time interval between a presynaptic input and the associated b-AP tends to be longer for a distant synapse. If synaptic efficacy is modified based on causal relations between presynaptic inputs and firings of a postsynaptic neuron, the dependence of the time window on location seems reasonable (Fig. 4(e)).

### The behavior of calcium propagation based on the chain activation of RyRs

We observed that the cytoplasmic calcium increase propagated along the dendrite by the chain activation of RyRs (Fig. 7). The speed of calcium propagation was several tens of micrometers per second and the propagation range was several hundred micrometers depending on the location of the induction site. Although our model did not consider the behavior of IP_3_R, these simulation results partly reproduce physiological data (Nakamura et al. 1999; Watanabe et al. 2006).

Whether calcium release occurs at neighboring sites other than the induction site or not depends on the timing of calcium influx via VDCC and calcium flux propagating from the induction site. Because propagation of a b-AP is much faster than diffusion of calcium concentration, calcium influx via VDCC activated by a b-AP occurred first and slightly increased calcium concentration in the ER. However, the pumped calcium ions slowly leaked as shown in Fig. 6(d). Thus, the calcium flux propagating from the induction site arrived later at a neighboring site. Therefore, it took more time to cause a calcium increase, in other words, it took longer to surpass the threshold-like C_cyt_-nullcline shown in Fig. 6. Consequently, along with the calcium propagation, the interval of the two fluxes was prolonged and temporal overlap of calcium increases between adjacent sites was reduced (Fig. 7(a, b)). As shown in the center and bottom part of Fig. 7(a, b), calcium increase at one site (red line) is followed by an increase at the adjacent site further away from the induction site (green line). The falling phase after the transient calcium increase (red-dashed line) was slowed down by calcium diffusion from the adjacent site where the concentration was still relatively high. As the overlap increased, the falling phase continued for a longer time due to this effect. In contrast, if the overlap was small, calcium concentration smoothly descended. Accordingly, closer to the induction site, the calcium increase tends to last longer. The comparison between Fig. 7(a) and 7(b) suggests that the continuation of calcium increase depends on the direction of the calcium propagation. This is because the calcium influx via VDCC attenuates along the dendrite (Fig. 3(b)); the duration of calcium increase becomes longer at a more distal site. This mechanism underlies the result that the attenuation of the integrated amount of calcium increase is relatively large at distal sites 200–235 μm away from the soma, as illustrated in Fig. 7(d). In addition, we observed that the calcium propagation from the dendritic branch tended to stop at the joint of the dendritic trunk and branch. In contrast, the propagation from trunk to branch was not prevented. This phenomenon was observed in physiological experiments (Nakamura et al. 2002). We also observed that the chain activation of RyR is suddenly cut at a certain point in the dendrite (Fig. 7, Fig. 9). These results suggest that the synaptic association is compartmentalized at the branching point; although collective synaptic change is localized within a compartment, it is able to affect synapses at branched compartments. This structural mechanism could contribute to the link between the local event of synaptic change and the formation of selectivity to synaptic input.

### Limitations of the modeling study

#### Exclusion of spine dynamics

It has been reported that RyRs are expressed not only in the dendritic shaft but also in spines of CA1 pyramidal neurons (Ellisman et al. 1990; Sharp et al. 1993). Furthermore, it has been shown that calcium store with RyR in a spine contributes to enhancement of calcium increase evoked by synaptic activation (Emptage et al. 1999). In general, however, many experimental studies have reported that RyRs have little influence on the calcium increase in a spine (Kovalchuk et al. 2000; Nevian and Sakmann 2006). The divergence of results likely derives from differences in experimental and/or cellular conditions. Therefore, we did not incorporate the calcium dynamics in a spine and focused on revealing the spatiotemporal calcium regulation at a cellular level by RyRs in the dendritic shaft in order to exclude uncertainty of calcium signaling in a spine.

#### Dependence of outcomes on responsiveness of calcium store

We observed that the dendritic calcium signaling consisted of two steps. The first step was an initiation of calcium release in the dendritic compartment of a homo-synapse, where the occurrence of calcium release depended on spike timings of pre- and postsynaptic neurons. However, the peak amount of calcium increase was almost constant for any timing within the spike-time window (Fig. 4(b)). The second step was the propagation of spike-like calcium increase along the dendritic shaft, at which there was no difference in the peak amount of calcium increase within the propagation range (Fig. 7(c)).

How does the RyR-calcium regulation affect the outcome of homo- and hetero-synaptic changes? The change in synaptic efficacy actually results from complex interactions of calcium signaling pathways (Berridge et al. 2000). Furthermore, whether synaptic efficacy is potentiated or depressed depends on the structure of a spine, subcellular distribution of endogenous buffer, and other factors. However, if we assume that a sufficient amount of released calcium ions diffuses from a dendritic shaft to a spine, and the calcium increase in a dendritic shaft can be regarded as the determinant of calcium-dependent synaptic change, the amount of the synaptic change caused at a homo-synapse would be almost constant irrespective of the pre- and postsynaptic spike timings. In addition, the calcium propagation regulated by RyRs would induce similar changes among hetero-synapses within the range of the calcium propagation. These predictions are based on the result that the profile of the calcium increase does not differ in intervals of pre- and postsynaptic spikes and locations in a dendrite as mentioned above. However, these predictions are partly inconsistent with the experimental evidence on the spike-timing-dependent synaptic plasticity at a homo-synapse (Bi and Poo 1998).

The characteristics mentioned above can be attributed to the all-or-none response of the incorporated RyR-sensitive calcium store model. In a CA1 pyramidal neuron, several different types of receptors (subtypes of RyRs and IP_3_Rs) express on ER (Sharp et al. 1993; Furuichi et al. 1994; Fitzpatrick et al. 2009). This response property does not depend on the type but crucially depends on many other factors, such as abundance and effect of intracellular calcium buffers, activity of calcium-dependent enzymes, and spatial formation of the receptor channels (Wehrens et al. 2004; Cheng and Lederer 2008). Even if a single channel exhibits an all-or-none response, the collective response of the channels practically depends on such factors. For example, because RyRs and IP_3_Rs form a cluster on ER, the spatial formation of the channels is one of the important factors. Furthermore, the actual activation of each channel is not deterministic but stochastic. In the case that the stochastic nature is strong, the overall response will be graded even if the response of each channel is all-or-none. We assessed the case where the collective response of RyRs could be well described by the deterministic model. In the case where the responsiveness of calcium store is of the graded type, the dependence of calcium release on spike intervals and the attenuation of calcium propagation would be more graded. Therefore, the distinct responsiveness would play different roles for spatiotemporal calcium dynamics; the all-or-none type would be responsible for detection of calcium influxes evoked by paired spikes and initiation of calcium increase, whereas the other type would contribute to adjusting the level of calcium concentration and assisting calcium release at neighboring sites.

#### Parameter dependence

As described above, we quantitatively assessed characteristics arising from the all-or-none response of the calcium store in the spatial propagation of calcium release. The characteristics were robust against changes in input conditions; the peak amount of calcium release was almost constant independent of the spike timing (Fig. 8(a, b), Fig. 9). In addition, at surrounding sites of the induction site, the level of calcium increase barely changed (Fig. 7(d)). However, RyR-regulated calcium propagation had a sensitive aspect: its chain-like activation sensitively continued or stopped depending on the properties of ER. Figure 9(a) implied a relationship between RyR-mediated calcium propagation and the spatial expression of RyR. Thus, assuming that the degrees of leaking and pumping functions of ER did not vary across different sites, and the rate of RyR (*v*_rel_ in Eq. (40)) reflected the expression level of RyR, the chain-like activation would transition to a failure condition by decreasing the expression level of RyRs. In addition, intracellular calcium flux dynamically fluctuates in vivo depending on molecular processes, neural activities, and movement of dendrite. If the spatial association of synaptic plasticity is brought about by the chain-like activation of RyRs, it would be reasonable to predict that the outcome is less robust against the fluctuations in such related processes. Therefore, RyR calcium regulation likely contributes to initiation rather than relay calcium propagation.

## References

[CR1] Allbritton N. L., Meyer T., Stryer L. (1992). Range of messenger action of calcium ion and inositol 1, 4, 5-trisphosphate. Science.

[CR2] Andrásfalvy B. K., Magee J. C. (2001). Distance-dependent increase in AMPA receptor number in the dendrites of adult hippocampal CA1 pyramidal neurons. Journal of Neuroscience.

[CR3] Berridge, M. J., Lipp, P., & Bootman, M. D. (2000). The versatility and universality of calcium signaling. Nature Reviews Molecular Cell Biology, 1(1), 11–21. 10.1038/35036035 11413485

[CR4] Bezprozvanny I., Watras J., Ehrlich B. E. (1991). Bell-shaped calcium-response curves of lns (l, 4, 5) P3-and calcium-gated channels from endoplasmic reticulum of cerebellum. Nature.

[CR5] Bi G. Q. (2002). Spatiotemporal specificity of synaptic plasticity: cellular rules and mechanisms. Biological Cybernetics.

[CR6] Bi G. Q., Poo M. M. (1998). Synaptic modifications in cultured hippocampal neurons: dependence on spike timing, synaptic strength, and postsynaptic cell type. Journal of Neuroscience.

[CR7] Bliss T. V. P., Collingridge G. L. (1993). A synaptic model of memory: long-term potentiation in the hippocampus. Nature.

[CR8] Bliss T. V. P., Lomo T. (1973). Long-lasting potentiation of synaptic transmission in the dentate area of the anaesthetized rabbit following stimulation of the perforant path. Journal of Physiology.

[CR9] Bradler J. E., Barrionuevo G. (1989). Long-term potentiation in hippocampal CA3 neurons: tetanized input regulates heterosynaptic efficacy. Synapse.

[CR10] Cheng H., Lederer W. J. (2008). Calcium sparks. Physiological Reviews.

[CR11] Colbran R. J., Brown A. M. (2004). Calcium/calmodulin-dependent protein kinase II and synaptic plasticity. Current Opinion in Neurobiology.

[CR12] Ellisman M. H., Deerinck T. J., Ouyang Y., Beck C. F., Tanksley S. J., Walton P. D. (1990). Identification and localization of ryanodine binding proteins in the avian central nervous system. Neuron.

[CR13] Emptage N., Bliss T. V. P., Fine A. (1999). Single synaptic events evoke NMDA receptor-mediated release of calcium from internal stores in hippocampal dendritic spines. Neuron.

[CR14] Fitzpatrick J. S., Hagenston A. M., Hertle D. N., Gipson K. E., Bertetto-D’Angelo L., Yeckel M. F. (2009). Inositol-1, 4, 5-trisphosphate receptor-mediated Ca2+ waves in pyramidal neuron dendrites propagate through hot spots and cold spots. Journal of Physiology.

[CR15] Fujii S., Matsumoto M., Igarashi K., Kato H., Mikoshiba K. (2000). Synaptic plasticity in hippocampal CA1 neurons of mice lacking type 1 inositol-1, 4, 5-trisphosphate receptors. Learning & Memory.

[CR16] Furuichi T., Furutama D., Hakamata Y., Nakai J., Takeshima H., Mikoshiba K. (1994). Multiple types of ryanodine receptor/Ca2+ release channels are differentially expressed in rabbit brain. Journal of Neuroscience.

[CR17] Futatsugi A., Kato K., Ogura H., Li S. T., Nagata E., Kuwajima G. (1999). Facilitation of NMDAR-independent LTP and spatial learning in mutant mice lacking ryanodine receptor type 3. Neuron.

[CR18] Gasparini S., Losonczy A., Chen X., Johnston D., Magee J. C. (2007). Associative pairing enhances action potential back-propagation in radial oblique branches of CA1 pyramidal neurons. Journal of Physiology.

[CR19] Golding N. L., Kath W. L., Spruston N. (2001). Dichotomy of action-potential backpropagation in CA1 pyramidal neuron dendrites. Journal of Neurophysiology.

[CR20] Graupner M., Brunel N. (2012). Calcium-based plasticity model explains sensitivity of synaptic changes to spike pattern, rate, and dendritic location. Proceedings of the National Academy of Sciences of the United States of America.

[CR21] Harris K. M., Stevens J. K. (1989). Dendritic spines of CA 1 pyramidal cells in the rat hippocampus: serial electron microscopy with reference to their biophysical characteristics. Journal of Neuroscience.

[CR22] Hertle D. N., Yeckel M. F. (2007). Distribution of inositol-1, 4, 5-trisphosphate receptor isotypes and ryanodine receptor isotypes during maturation of the rat hippocampus. Neuroscience.

[CR23] Hirsch J. C., Barrionuevo G., Crepel F. (1992). Homo- and heterosynaptic changes in efficacy are expressed in prefrontal neurons: an in vitro study in the rat. Synapse.

[CR24] Hulme S. R., Jones O. D., Ireland D. R., Abraham W. C. (2012). Calcium-dependent but action potential-independent BCM-like metaplasticity in the hippocampus. Journal of Neuroscience.

[CR25] Johnston D., Christie B. R., Frick A., Gray R., Hoffman D. A., Schexnayder L. K. (2003). Active dendrites, potassium channels and synaptic plasticity. Philosophical Transactions of the Royal Society of London. Series B: Biological Sciences.

[CR26] Jung, H. Y., Mickus, T., & Spruston, N. (1997). Prolonged sodium channel inactivation contributes to dendritic action potential attenuation in hippocampal pyramidal neurons. Journal of Neuroscience, 17(17), 6639–6647. 10.1523/JNEUROSCI.17-17-06639.1997 PMC6573150 9254676

[CR27] Katz L. C., Shatz C. J. (1996). Synaptic activity and the construction of cortical circuits. Science.

[CR28] Keizer J., Levine L. (1996). Ryanodine receptor adaptation and Ca2+ (−) induced Ca2+ release dependent Ca2+ oscillations. Biophysical Journal.

[CR29] Khodakhah K., Armstrong C. M. (1997). Inositol trisphosphate and ryanodine receptors share a common functional Ca2+ pool in cerebellar Purkinje neurons. Biophysical Journal.

[CR30] Kovalchuk Y., Eilers J., Lisman J., Konnerth A. (2000). NMDA receptor-mediated subthreshold Ca2+ signals in spines of hippocampal neurons. Journal Neuroscience.

[CR31] Lynch G. S., Dunwiddie T., Gribkoff V. (1977). Heterosynaptic depression: a postsynaptic correlate of long-term potentiation. Nature.

[CR32] Markram H., Lubke J., Frotscher M., Sakmann B. (1997). Regulation of synaptic efficacy by coincidence of postsynaptic APs and EPSPs. Science.

[CR33] Martin S. J., Grimwood P. D., Morris R. G. M. (2000). Synaptic plasticity and memory: an evaluation of the hypothesis. Annual Review of Neuroscience.

[CR34] Migliore M., Hoffman D., Magee J., Johnston D. (1999). Role of an a-type K+ conductance in the back-propagation of action potentials in the dendrites of hippocampal pyramidal neurons. Journal of Computational Neuroscience.

[CR35] Munton R. P., Vizi S., Mansuy I. M. (2004). The role of protein phosphatase-1 in the modulation of synaptic and structural plasticity. FEBS Letters.

[CR36] Nakamura T., Barbara J. G., Nakamura K., Ross W. N. (1999). Synergistic release of Ca2+ from IP3-sensitive stores evoked by synaptic activation of mGluRs paired with backpropagating action potentials. Neuron.

[CR37] Nakamura T., Lasser-Ross N., Nakamura K., Ross W. N. (2002). Spatial segregation and interaction of calcium signalling mechanisms in rat hippocampal CA1 pyramidal neurons. Journal of Physiology.

[CR38] Nevian T., Sakmann B. (2006). Spine Ca2+ signaling in spike-timing-dependent plasticity. Journal of Neuroscience.

[CR39] Nishiyama M., Hong K., Mikoshiba K., Poo M., Kato K. (2000). Calcium stores regulate the polarity and input specificity of synaptic modification. Nature.

[CR40] Parekh A. B., Putney J. W. (2005). Store-operated calcium channels. Physiological Reviews.

[CR41] Pettit D. L., Augustine G. J. (2000). Distribution of functional glutamate and GABA receptors on hippocampal pyramidal cells and interneurons. Journal of Neurophysiology.

[CR42] Poirazi P., Brannon T., Mel B. W. (2003). Arithmetic of subthreshold synaptic summation in a model CA1 pyramidal cell. Neuron.

[CR43] Raymond C. R., Redman S. J. (2002). Different calcium sources are narrowly tuned to the induction of different forms of LTP. Journal of Neurophysiology.

[CR44] Reyes M., Stanton P. K. (1996). Induction of hippocampal long-term depression requires release of Ca2+ from separate presynaptic and postsynaptic intracellular stores. Journal of Neuroscience.

[CR45] Rose C. R., Konnerth A. (2001). Stores not just for storage: intracellular calcium release and synaptic plasticity. Neuron.

[CR46] Royer S., Paré D. (2003). Conservation of total synaptic weight through balanced synaptic depression and potentiation. Nature.

[CR47] Sabatini B. L., Oertner T. G., Svoboda K. (2002). The life cycle of Ca(2+) ions in dendritic spines. Neuron.

[CR48] Seymour-Laurent K. J., Barish M. E. (1995). Inositol 1,4,5-tris-phosphate and ryanodine receptor distributions and patterns of acetylcholine- and caffeine-induced calcium release in cultured mouse hippocampal neurons. Journal of Neuroscience.

[CR49] Sharp A. H., McPherson P. S., Dawson T. M., Aoki C., Campbell K. P., Snyder S. H. (1993). Differential immunohistochemical localization of inositol 1, 4, 5-trisphosphate-and ryanodine-sensitive Ca2+ release channels in rat brain. Journal of Neuroscience.

[CR50] Sjöström P. J, Turrigiano G. G., Nelson S. B. (2001). Rate, timing, and cooperativity jointly determine cortical synaptic plasticity. Neuron.

[CR51] Stuart G., Spruston N. (1998). Determinants of voltage attenuation in neocortical pyramidal neuron dendrites. Journal of Neuroscience.

[CR52] Takahashi N., Kitamura K., Matsuo N., Mayford M., Kano M., Matsuki N. (2012). Locally synchronized synaptic inputs. Science.

[CR53] Usachev Y. M., Thayer S. A. (1997). All-or-none Ca2+ release from intracellular stores triggered by Ca2+ influx through voltage-gated Ca2+ channels in rat sensory neurons. Journal of Neuroscience.

[CR54] Watanabe S., Hong M., Lasser-Ross N., Ross W. N. (2006). Modulation of calcium wave propagation in the dendrites and to the soma of rat hippocampal pyramidal neurons. Journal of Physiology.

[CR55] Wehrens X. H., Lehnart S. E., Reiken S. R., Marks A. R. (2004). Ca2+/calmodulin-dependent protein kinase II phosphorylation regulates the cardiac ryanodine receptor. Circulation Research.

[CR56] Zador A., Koch C., Brown T. H. (1990). Biophysical model of a hebbian synapse. Proceedings of the National Academy of Sciences of the United States of America.

